# A Psychophysiological Model of Firearms Training in Police Officers: A Virtual Reality Experiment for Biocybernetic Adaptation

**DOI:** 10.3389/fpsyg.2020.00683

**Published:** 2020-04-16

**Authors:** John E. Muñoz, Luis Quintero, Chad L. Stephens, Alan T. Pope

**Affiliations:** ^1^Department of Systems Design Engineering, University of Waterloo, Waterloo, ON, Canada; ^2^Department of Computer and Systems Sciences, Stockholm University, Stockholm, Sweden; ^3^Langley Research Center, National Aeronautics and Space Administration, Hampton, VA, United States; ^4^Learning Engagement Technologies, Poquoson, VA, United States

**Keywords:** biocybernetic adaptation, virtual reality, psychophysiological model, electroencephalography, heart rate variability, simulation, firearms training

## Abstract

Crucial elements for police firearms training include mastering very specific psychophysiological responses associated with controlled breathing while shooting. Under high-stress situations, the shooter is affected by responses of the sympathetic nervous system that can impact respiration. This research focuses on how frontal oscillatory brainwaves and cardiovascular responses of trained police officers (*N* = 10) are affected during a virtual reality (VR) firearms training routine. We present data from an experimental study wherein shooters were interacting in a VR-based training simulator designed to elicit psychophysiological changes under easy, moderate and frustrating difficulties. Outcome measures in this experiment include electroencephalographic and heart rate variability (HRV) parameters, as well as performance metrics from the VR simulator. Results revealed that specific frontal areas of the brain elicited different responses during resting states when compared with active shooting in the VR simulator. Moreover, sympathetic signatures were found in the HRV parameters (both time and frequency) reflecting similar differences. Based on the experimental findings, we propose a psychophysiological model to aid the design of a biocybernetic adaptation layer that creates real-time modulations in simulation difficulty based on targeted physiological responses.

## Introduction

Burdea and Coiffet (2003) identified “traditional” areas of application of virtual reality (VR) as medicine, education, arts, entertainment, and the military as well as “emerging” areas of manufacturing, robotics and data visualization, training being a particular focus area in many of these applications. For use in training, VR environments provide advantages over physical training environments. VR training in medical surgery contexts has shown performance improvements beyond traditional training approaches (Lehmann et al., 2005). The use of immersive training systems taps into gross and fine motor skill acquisition, maintenance, and expert-level performance (Faria et al., 2018). Firearms training is considered an especially appropriate setting for VR technology deployment. Due to the safety concerns associated with live fire weapons training, the United States Department of Defense places a high value on the potential use of VR environments for firearms training for service members who are impaired by polytrauma (Oliver et al., 2019).

Under high-stress situations, the shooter is affected by responses of the sympathetic nervous system that can impact respiration. Relaxing the body and keeping a natural breathing pattern have been identified as major components of firearms training (Johnson, 2007). A training guide well known to the professional police officer trainer community asserts that particular sympathetic responses are desired during military fighting situations [e.g., 100–115 BPMs for heart rate (HR) levels] (Mason, 1998). According to marksmanship training guidelines (Johnson, 2007), an important factor that needs to be trained in overall marksmanship scenarios is shooting during the natural respiratory pause. Since the lack of oxygen might disturb the performance of cognitive skills and visual acuity, training autogenic breathing (autonomic self-regulation training) is considered as a main component of firearms training (Sajnog, 2013), which will calm the body and keep a natural breathing pattern. In order to maintain both body and mind collected, proper oxygenation could be provided by controlling the optimal respiration pace and peripheral responses such as HR and HR variability (HRV).

To support investigation of physiological self-regulation in police officers while training in a target-shooting scenario, a fully immersive, VR-based training simulator called Biocyber Physical Simulator (BioPhyS) for firearms training has been created (Muñoz et al., 2016b). BioPhyS is an example of a system that is capable of employing a form of physiological computing known as biocybernetic adaptation wherein real-time data from the brain and body is used by a control loop to adapt the user interface (Ewing et al., 2016). The design of the BioPhyS system relies on investigations of physiological responses associated with the psychophysiological responses of firearm trainees during a targeted state associated with concentration and calmness.

The research reported here focuses on how frontal oscillatory brainwaves and cardiovascular responses of experienced police officers are affected during a head-mounted display-VR (HMD-VR) training routine. This article uses insights from a previously reported research study (see Muñoz et al., 2019, for more details) to investigate how brain and heart of police officers react to a simulated training for firearms use. Particularly, this research is focused on:

• *Quantifying the neurophysiological and cardiovascular responses* of a group of trained police officers while interacting with a controlled and immersive virtual simulation of shooting practice.
• Investigating *perceived motion sickness and user experience* of the police officers after being immersed in a VR simulator for firearms training while wearing physiological sensors.
• Establishing a *psychophysiological model of firearm training for real police officers* while using immersive HMD-VR and non-intrusive physiological sensors. The model considers specific metrics from electroencephalographic (EEG) and cardiovascular sensors (e.g., HR and HRV) and how those metrics vary when the difficulty in the simulator was modulated.
• Illustrating a *streamlined pipeline for the integration of physiological adaptation into VR* simulators using novel tools such as biocybernetic software technologies and biofeedback design elements.

## Related Work

Lele (2013) points to the quality of immersion as the basis for a VR system’s usefulness. Psychophysiological measurement has increasingly been incorporated into research using HMD-VR setups (Pugnetti et al., 2001), particularly in studies examining presence and immersion. Cortical, as well as cardiovascular measures, have relevance for characterizing the psychophysiological response of individuals experiencing VR environments.

In one study, service members with polytrauma demonstrated improved accuracy and precision following VR-based firearm training (Oliver et al., 2019), due to its capability to measure metrics that are not measured in a traditional qualification course, and allowing instructors to focus on other aspects to enhance shooter precision such as posture, sight alignment, or the elimination of bad habits. The United States Navy has developed the tactically reconfigurable artificial combat enhanced reality (TRACER) system to train sailors for combat. TRACER is extolled as a dynamic, engaging and less predictable virtual training environment (Iatsyshyn et al., 2019). In an analysis of the development status and characteristics of VR technology in China (Zhang et al., 2019), it is asserted that VR be beneficial in improving the psychological wellbeing of military personnel and help soldiers to adapt to various war environments. In this regard, exposure therapy based on VR has been extensively showed as an efficacious treatment for active-duty army soldiers with post-traumatic stress disorder (Rizzo and Shilling, 2017). EEG has been employed in investigations of various cognitive variables in 3D virtual learning environments. Frontal alpha EEG was recorded in a study of its role in attentional control; the study also demonstrated the importance of the immersion and engagement afforded by a 3D virtual learning environment (Berger and Davelaar, 2018). In a study of the effect of competition, while shooters were immersed in a virtual environment representing a shooting range, changes in alpha oscillatory activity were found during aiming that were associated with better performance (Pereira et al., 2018). In a previous study by the authors (Muñoz et al., 2016b), frontal alpha activity (as well as delta) was found to be a brainwave pattern that allows differentiation between baseline and active shooting states in a VR simulator. Increases in alpha EEG activity have also been associated with subjects who performed well in spatial navigation tasks in a VR environment (Pugnetti et al., 1996).

Virtual reality experiences can also affect the cardiovascular responses of users. A significant difference in HR values was found during 5-min long interactions with a VR experience that required users to perform simple manual tasks involving the arrangement of virtual elements with multiple shapes (Malińska et al., 2015). A recent study (Marín-Morales et al., 2019) employed analyses of EEG and electrocardiographic (ECG) signals to explore differences in the emotional reactions (arousal and valence dimensions) between an exploration in a real-museum and its VR representation, and demonstrated high accuracy of a machine learning model in classifying the nature of a stimuli as real or virtual.

Machine learning approaches have also been used in psychophysiological studies. Specifically, the use of supervised classifiers [e.g., linear discriminant analysis (LDA) and support vector machines (SVM)] to categorize emotional states and create a model for audio-visual or game difficulty adaptation (Novak et al., 2012). Other studies have utilized multilayer perceptron to classify anxiety, boredom, and flow states to compare the effects of mental-state adaptation and performance-based adaptation in a shooting desktop-based game (Alves et al., 2018). A custom-made version of the conventional Tetris was adapted to create real-time adjustments of the game difficulty in three levels by using a SVM classifier that processed signals from skin response, blood volume and EEG (Chanel et al., 2011). Furthermore, learning-based classifiers have also been used to detect high and low anxiety in drivers from ECG and accelerometer data (Dobbins and Fairclough, 2018), or to create a virtual driving platform to maximize engagement in people with autism spectrum disorder (Bian et al., 2019). Applications for airplane pilots used classifiers to identify features from EEG and skin response signals that can model the users in scenarios of attention-related human performance limiting states (Harrivel et al., 2016) or to find relationships of cardiovascular features with psychophysiological stress while performing piloting maneuvers (Hanakova et al., 2017). To the best of our knowledge, this is the first project that aims at characterizing psychophysiological responses of police officers on duty for designing biocybernetic loops in VR firearms training.

## Virtual Reality for Firearms Training

In this section, we describe the design and development of a fully immersive, HMD-VR based simulator for firearms shooting training as well as briefly introduce a software tool, the Biocybernetic Loop (BL) Engine, used to integrate physiological intelligence to the VR simulator.

### Biocyber Physical Simulator System for Firearms Training

**FIGURE 1:**
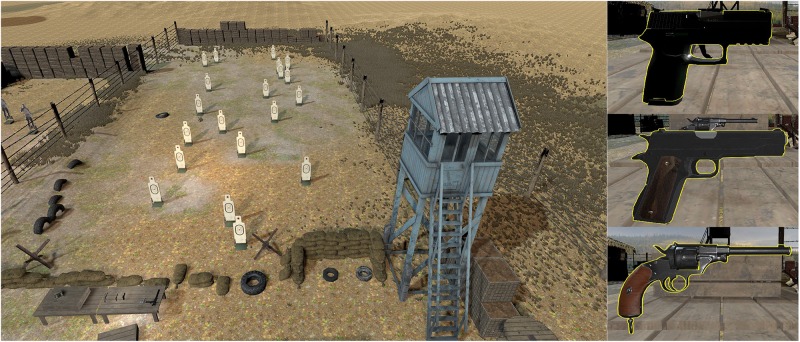
Screenshots of the BioPhyS virtual simulator created in VR. **Left:** top-view of the shooting range with the targets placed randomly and a table with the guns. **Right:** view of the grabbable weapons that can be shot during the simulation.

The BioPhyS contains an outdoor military target-shooting range with representative props such as cable reels, wooden tables, barricades, weapons, and water towers (see Figure 1, left). Twenty targets were laid out at different distances from the shooting point; each target moves along a horizontal track. Three weapons were used for training: The Pistols M1911 and SIG Sauer P250, and the Reichsrevolver M1879 (see Figure 1, right), each with different impact force on the targets.

A setup panel called Wizard of Oz (WoZ) was designed to allow trainers to modify in real-time the conditions of the simulation; it also provided a controlled environment to study the participants’ responses and behaviors when exposed to specific stressors. The set of variables was defined to create physiological modulation and were grouped according to its effect on the scenario, as shown in Table 1.

**TABLE 1:** List of simulation variables carefully defined to create the physiological modulation in the BioPhyS.

**Simulation effect**	**Simulation variable**	**Range**
Targets	Number of targets	3–20 (targets)
	Target size	0.3–3 (units)
	Target speed (horizontal)	0–5 (m/s)
	Target hardness	1–20 (units)
Simulation environment	Day light	0–10 (units)
	Rain intensity	0–1 (units)

BioPhyS can be used with wireless HTC Vive controllers and an air pistol gun adapted with the HTC Vive Tracker to handle and shoot the weapon. The touchpad of the controllers offers teleportation to navigate around the shooting range, the lateral buttons are used to grab the virtual gun, and the trigger is used for shooting.

### Physiological Intelligence and the Biocybernetic Loop Engine

Conventional methods to integrate physiological sensing technologies into games and VR applications entail the development of specific software clients that stream data collected from the sensors and capturers able to read the data packages directly in game engines such as Unity3D (Muñoz et al., 2016a). Additionally, scripts for signal processing and data analysis are required to create truly intelligent algorithms able to finally integrate biocybernetic adaptation. To streamline the process, the BL Engine is a software tool that acts as a middle layer facilitating the integration of physiological intelligence to games and VR applications developed in Unity3D (Muñoz et al., 2017b). By allowing (i) communication with multiple physiological sensors, (ii) a drag-and-drop console to create adaptive rules, and (iii) specialized scripts to communicate applications and modify variables in real-time; the BL Engine is a software tool to simplify the integration of biocybernetic adaptation in VR projects. This article outlines our initial stage of designing the biocybernetic adaptation layer after carrying out a physiological characterization study in police officers while interacting with the BioPhyS with controlled difficulty levels.

## Psychophysiological Characterization Study With Police Officers

The BioPhyS was used to conduct a controlled study with police officers using a repeated measures design, to understand the cardiovascular and neurophysiological responses under different simulation difficulties.

### System Setup

**FIGURE 2:**
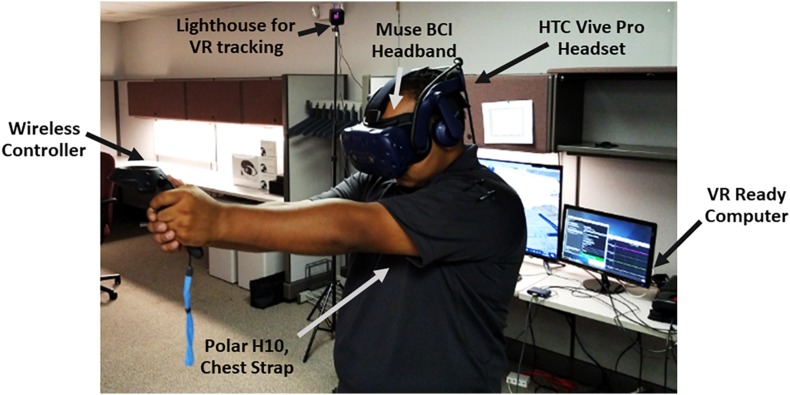
System setup for the study describing the equipment used.

A room-scale tracking system of the VR headset HTC Vive Pro was used to provide a fully immersive experience. The users were able to walk in an area of up to 12 square meters (maximum 5 m between both tracking lighthouses) and interact with the virtual environment by using one wireless controller. A VR One MSI backpack (VR ready computer) computer to run BioPhyS, and an additional screen was used to configure the scenarios and to mirror the VR simulation (see Figure 2).

### Participants

Ten police officers from the police division of Hampton city (VA, United States) were recruited for the study. Participants were mostly males (nine males and one female) with ages from 21 to 43 years old. The participation in the study was advertised by the police department as voluntary and the inclusion criteria was having past experience with real target shooting. The experiment was described as a playtest and the details were given to the participants before starting; every police officer also signed informed consent. Table 2 summarizes the characteristics of the police officers who participated in the study.

**TABLE 2:** Police officer’s demographics and firearm training.

**User ID**	**Age**	**Gender**	**Target shooting experience**	**Military veteran**	**Time as LEO**	**Firearms qualifications requirement**	**Specialized weapons training**
01	37	Male	>3 years	No	17 years	Bi-Annual	SWAT, Firearms Instructor
02	37	Male	>3 years	Yes	3 years	Annual, Bi-Annual	None
03	43	Male	>3 years	Yes	In Academy	Annual, Bi-Annual	None
04	21	Male	<1 year	No	In Academy	Bi-Annual	None
05	32	Male	>3 years	No	11 years	Annual, Bi-Annual	Firearms instructor, SWAT, Patrol rifle
06	26	Female	>3 years	No	3 years	Bi-Annual	Firearms instructor
07	31	Male	>3 years	Yes	In Academy	Bi-Annual	None
08	33	Male	>3 years	No	15 years	Bi-Annual	Firearms instructor, SWAT
09	27	Male	<1 year	No	In Academy	Bi-Annual	None
10	23	Male	<1 year	No	In Academy	Bi-Annual	None

*LEO, Law Enforcement Officer; SWAT, special weapons and tactics.*

### Physiological Metrics

Police officers were wearing both wearable EEG and HR monitors during the training session using the HMD-VR system. The signals were synchronously recorded on a secondary computer.

#### Electroencephalography

Brainwave activity was recorded using the wearable headband Muse BCI. The sensor includes four EEG electrodes in the TP9, Fp1, Fp2, and TP10 channel positions following the 10–20 standards. The device generates samples at a frequency of 500 Hz and contains proprietary algorithms that compute relevant parameters to quantify brain activity patterns such as the oscillatory rhythms (Sanei and Chambers, 2013) delta (δ, 1–4 Hz), alpha (α, 8–12 Hz), beta (β, 12–30 Hz), theta (θ, 4–8 Hz), and gamma (γ, 30–100 Hz). The proprietary software Muse Lab^1^ was used to compute the power spectral density (PSD) of the EEG raw data for each channel for a frequency range from 0 to 110 Hz, using a Hamming window with a window-length of 256 samples and 90% overlap. The EEG metrics that were more relevant for exploration were:

• Absolute Bandpowers: which use a logarithmic function with the sum of the PSD of the EEG data in a specific frequency range. The absolute α, β, ∂, θ, and γ bandpowers were computed for the frontal electrodes of the Muse BCI sensor.
• EEG indexes: three different indexes were explored considering previous studies (Kamzanova et al., 2011; Fairclough and Gilleade, 2012; McMahan et al., 2015). First, the Engagement index was computed by using the ratio of beta to alpha + theta. The frontal asymmetry index was computed by subtracting the Alpha in Fp2 (right hemisphere) with the Alpha in Fp1 (left hemisphere). Finally, the Theta/Beta ratio was also computed to see the neurophysiological responses in those two bandpowers.

#### Cardiovascular

The chest strap sensor Polar H10 was used to record the cardiac responses of police officers. It includes built-in algorithms that were used to calculate ECG parameters needed for the HRV analysis. The main variable computed is the R-to-R intervals (RRI) with units of 1/1024 seconds.^2^ HR and RRIs are broadcast by the sensor and saved locally using a custom-made client running on Windows and based on a Bluetooth Low Energy Windows API. The PhysioLab toolbox (Muñoz et al., 2017a) was used to compute both time and frequency domain HRV parameters. Extracted features include the standard deviation of the RRIs (SDNN) and the root mean square of successive differences (RMSSD) values from the time domain. Similarly, frequency domain parameters included high frequency (HF, 0.15–0.40 Hz), low frequency (LF, 0.04–0.15 Hz), and very low frequency (VLF, 0.0033–0.04 Hz), which were extracted from the PSD of the RRI signal. The PSD is computed by using a Welch estimator with a Hanning window, and spectrum components are averaged by an area-under-the-curve approach. The polar chest strap has shown acceptable performance in calculating HR and RRI measurements under different scenarios including non-resting situations (Plews et al., 2017).

### Other Measurements

#### Simulation Performance

The BioPhyS computed the participants’ performance during firearm shooting training. It recorded specific simulation variables such as the total amount of shot bullets, number of destroyed targets, and headshots. The shooting performance metric was defined as the ratio between the number of destroyed targets and the shot bullets.

#### Simulation Sickness

The level of motion sickness produced during interaction with the VR system was evaluated using the Simulator Sickness Questionnaire (SSQ; Kennedy et al., 1993). It assesses total severity of simulation sickness supported by three main symptom clusters called oculomotor (eyestrain, difficulty focusing, blurred vision, and headache), disorientation (dizziness and vertigo), and nausea (nausea, stomach awareness, increased salivation, and burping).

#### Post-session Subjective Interview

Police officers were briefly interviewed at the end of the session to gather their reactions in terms of (i) overall user experience, (ii) pros and (iii) cons of training with the BioPhyS, and (iv) envisioned improvements.

### Experimental Protocol

#### Training Scenarios

The training scenarios were jointly designed together with a military veteran of the research team. The training protocol included three difficulty modes: easy, medium, and hard; each of them lasting 3 min. The easy configuration laid out 10 static targets randomly distributed in the 10 first horizontal tracks of the training scenario. The medium setup used 10 moving targets at a speed of 0.5 m/s. The hard scenario used 20 targets moving at 1 m/s and it was specifically designed to provoke frustration. Both the target size and hardness were maintained constant across the difficult levels. The same weapon Reichsrevolver was used during the experiment setup with a shooting power equals to the hardness of the target, thus targets were instantly destroyed with one shot. Although unrealistic, the revolver was preferred considering its slow response, thus aiding a more careful aiming and shooting instead of less mentally and physically prepared training. A baseline condition was used to record physiological signals from the police officers during a passive stand-up situation, wearing the VR headset and physiological sensors, and holding the virtual weapon without shooting or interacting with the virtual environment.

#### Procedure

The study protocol was reviewed for ethical treatment of human subjects and approved by the Office of the Chief of Police of the City of Hampton, VA, serving as the research ethics committee. All police officer subjects volunteered to participate and gave written informed consent in accordance with the Declaration of Helsinki. The experiment lasted around 40 min per participant, including questionnaires, the connection of sensors and interaction. The informed consent was signed at the beginning of the experiment together with a short demographics form. Then, the sensors were connected to the police officer’s forehead and chest; the VR headset was also worn and participants were given the instruction to shoot some targets before starting to verify that the protocol was understood properly. Baseline physiological measurements were taken for 3 min, emphasizing that users needed to avoid any facial expression, such as speaking or visual navigation while moving the head. During the active shooting moments, police officers were instructed to also avoid squinting the eyes for aiming in order to minimize signal artifacts in the EEG sensor caused by facial movements. The easy-medium-hard scenarios were manually set up by the researcher, the participants interacted with them for a 2-min resting period between sessions. Finally, users filled out the SSQ and the short post-session interview.

#### Data Analysis

Collected physiological data was processed offline using MatLab (v2013b). Individual cardiovascular and EEG parameters were computed and averaged for statistical analysis. EEG bandpowers and index analysis initially explored frontal Fp1 and Fp2 electrodes separately; however, frontal lobe activity was ultimately weighted by averaging the contribution of both electrodes. Data normality was checked using Kolmogorov-Smirnoff tests. Data with normal distribution were statistically analyzed using parametric tests, whereas non-parametric tests were used for non-normal distributions to determine the influence of the simulation difficulty as the main effect. *Posthoc* analysis using Bonferroni correction was used to follow up the findings.

## Results

### Physiological Responses

#### Electroencephalography

**FIGURE 3:**
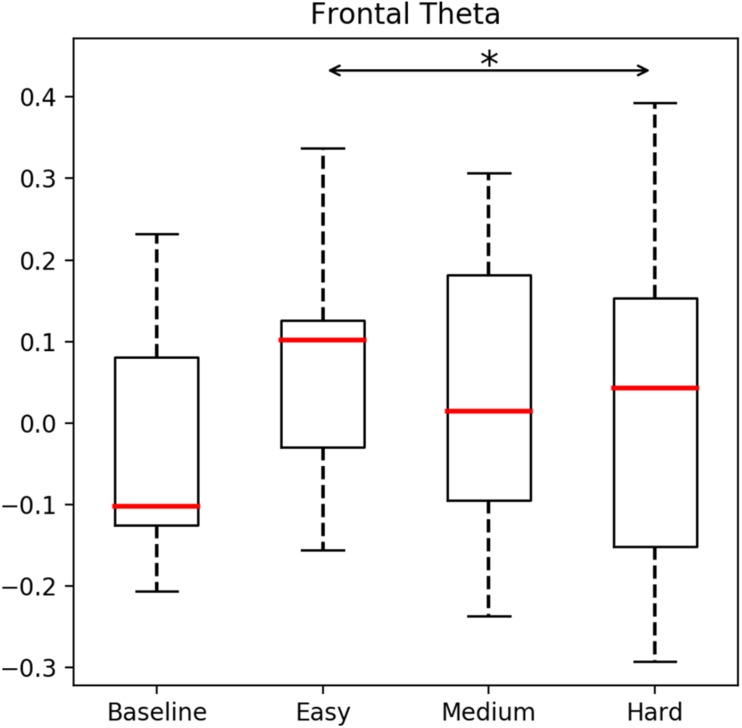
Theta brainwave patterns analyzed for the baseline, easy, medium, and hard difficulties in the BioPhyS. Asterisk (^∗^) denotes significant results following Wilcoxon tests.

Electroencephalography data recorded from the frontal lobe using the Fp1 and Fp2 electrodes of the Muse BCI system revealed significant brain activity patterns associated with the different difficulty levels used in the BioPhyS. Two specific EEG patterns showed significant results. Firstly, the frontal theta levels (θ), χ^2^(3) = 10.21, *p* < 0.05, showed significant changes across simulation difficulty levels. Wilcoxon tests were used to follow up initial findings while Bonferroni adjustments were applied, so all effects are reported at a 0.0125 level of significance. Results revealed that frontal theta (θ) activity differed significantly from *easy* to *hard* difficulty levels (*Z* = −1.58, *p* = 0.009) for the police officers (see Figure 3). Secondly, although the theta/beta ratio was significantly influenced by the simulation difficulty factor, χ^2^(3) = 10.69, *p* < 0.05, non-significant differences were found with the Wilcoxon test.

#### Cardiovascular Responses

**FIGURE 4:**
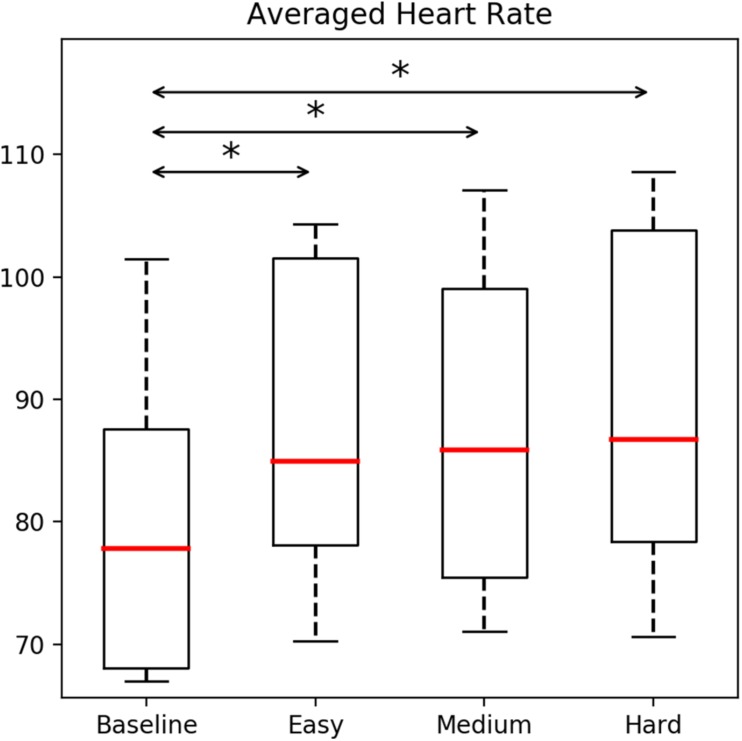
Heart rate (HR) levels affected during the training with the BioPhyS. Asterisk (^∗^) denotes significant differences following Wilcoxon tests.

Cardiovascular responses were quantified via HR and HRV parameters. HRs of police officers were significantly affected by the simulation difficulty, χ^2^(3) = 18.84, *p* < 0.05. *Post hoc* tests (Wilcoxon with Bonferroni) showed significant differences between the baseline measurements of HR compared with the *easy* (*Z* = −2.80, *p* = 0.005), *medium* (*Z* = −2.80, *p* = 0.005), and *hard* (*Z* = −2.80, *p* = 0.005), difficulty levels (see Figure 4).

The HRV analysis showed similar results for both time and frequency domains. RMSSD, χ^2^(3) = 18.48, *p* < 0.05, and VLF, χ^2^(3) = 18.84, *p* < 0.05; values were significantly affected by the simulation difficulty. Particularly the differences between baseline and *easy* (*Z* = −2.80, *p* = 0.005), *medium* (*Z* = −2.80, *p* = 0.005), and *hard* (*Z* = −2.80, *p* = 0.005) were significant in the *post hoc* analysis.

#### Simulation Performance

Simulation (or shooting) performance was computed as the ratio between the number of destroyed targets and the shot bullets. Simulation difficulty significantly influenced the shooting performance of police officers, χ^2^(3) = 18.84, *p* < 0.05, revealing 64, 46, and 24% performance for the *easy*, *medium*, and *hard* difficulties, respectively.

#### Simulation Sickness

Simulation sickness was measured immediately following the experience by asking police officers about their physical and cognitive status using the SSQ. Data from one of the users was discarded due to recording errors. Test subjects reported eight or fewer SSQ symptoms which is categorized as minimal symptoms, indicating the VR experience did not impact users’ operation of the system (Kennedy et al., 2001; Saredakis et al., 2020). Further analysis of the SSQ was conducted and cut-off scores were defined based on the 75th percentile of the calibration sample, to represent the majority of users in the test sample, to detect whether users were adversely affected by exposure to the virtual reality scenario (Kennedy et al., 1993). The reference scores are 15 for total severity, the thresholds for the subscales are 9.5 for nausea, 15.2 for oculomotor, and 0 for disorientation. Results revealed that three police officers were having sickness score above the threshold in all four aspects, and a total of six participants had high scores in the subscales of nausea and disorientation. Eye strain and difficulty focusing were reported by half of the police officers at the end of the training, although they never reached severe intensities.

**FIGURE 5:**
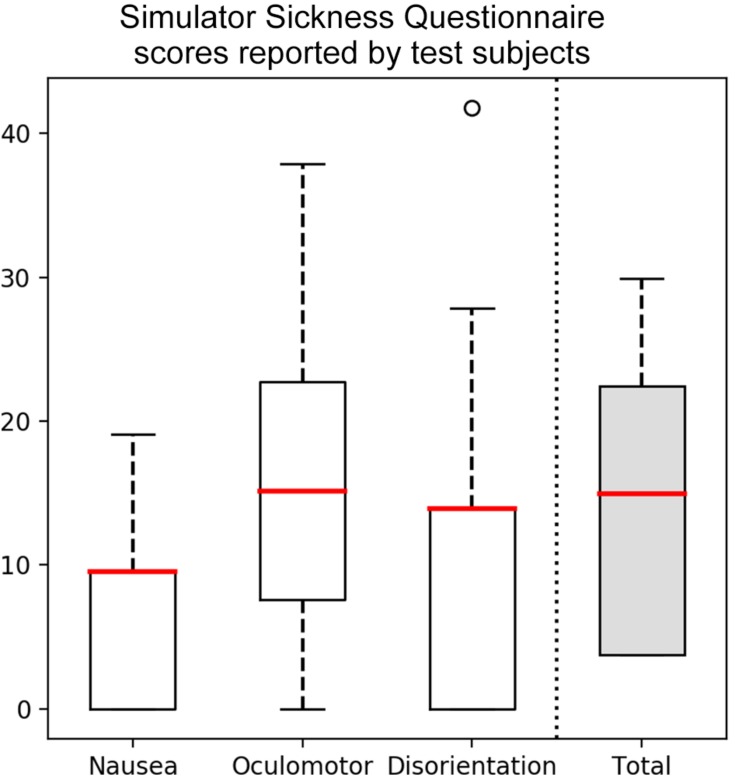
Simulator Sickness Questionnaire (SSQ) scores reported by test subjects for nausea (M = 8.5, SD = 7.4), oculomotor (M = 16.8, SD = 12.4), disorientation (M = 13.9, SD = 13.9), and total severity (M = 15.4, SD = 11.1).

Additionally, the mean and standard deviation were computed for the three distinct symptom clusters and the total score for the SSQ, and the relative severity was calculated by comparison with the nine calibration simulators utilized in the questionnaire. Results, depicted in boxplot format in Figure 5, indicate SSQ total severity (M = 15.4, SD = 11.1), nausea (M = 8.5, SD = 7.4), oculomotor (M = 16.8, SD = 12.4) and disorientation (M = 13.9, SD = 13.9) that fall between the first and second simulators with higher sickness. Therefore examination of the data and further development of the simulator is necessary to reduce effects of the VR simulation used in this study on users.

### Subjective Interview

Overall user experience was described positively by all police officers participating in the study. Two participants emphasized that the simulation made them focus on breathing and concentrate on remaining calm while shooting. Five participants described the experience as “good” and “cool” while highlighting their enthusiasm for trying VR for the first time. One subject mentioned that “graphics were really good” while others mentioned being “stressful as things became more difficult.” As advantages (pros) of the simulation system, three participants liked the simplicity of the setup, mentioning that the sensors were minimally invasive and comfortable. Three participants highlighted the realism of the simulation and the accuracy of the motion tracking. The possibility to personalize the training scenarios was mentioned by one of the users as the most remarkable advantage of the system. As disadvantages (cons) of the simulation system, five police officers mentioned being concerned about the sights and the aiming issues resulting from not allowing them to squint their eyes. Additionally, three officers reported some blurriness in the HMD. Three participants also mentioned mapping problems between the controllers and the virtual weapon since “the controller not as much like a weapon as would like.” Finally, police officers proposed several features that can be implemented to improve the system, from which we highlight the following ones: (i) change controllers for air pistols (or more realistic devices), (ii) allow users to see sights of the weapon to improve shooting accuracy, and (iii) integrate scenario-driven interactions.

Finally, the four police officers trained as firearms instructors endorsed the potential of the system for firearm training, the simplicity of the setup including the HMD-VR headset and physiological sensors and the training scenario personalization.

## Theoretical Model to Integrate Biocybernetic Adaptation

### Psychophysiological Model

**FIGURE 6:**
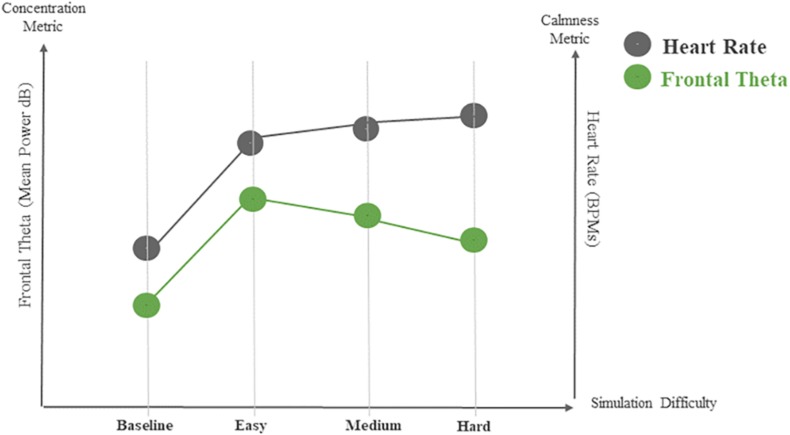
Psychophysiological model for the BioPhyS considering EEG and HR data.

A psychophysiological model based on empirical findings under various levels of challenge is an integral part of the design of a biocybernetic adaptation layer that creates real-time modulations in simulation difficulty based on targeted physiological responses. The resulting psychophysiological model derived for the trained police officer population in this study is used to inform the biocybernetic adaptation approach that can be integrated into the simulation system by using the BL Engine. With the BioPhyS system, this model is developed by characterizing the difference between resting states when compared with active shooting states in a VR simulator in both brain responses and sympathetic signatures. This characterization helps specify the values of physiological variables to be targeted in the biocybernetic adaptation system as well as serve as a control measure for assessing the effects of adding a biocybernetic adaptation layer to a VR training system. Considering the results from the characterization study, we created a psychophysiological model (see Figure 6) that reveals opportunities to integrate biocybernetic adaptation in the BioPhyS using the police officers’ data.

### Preliminary Computational Model

**FIGURE 7:**
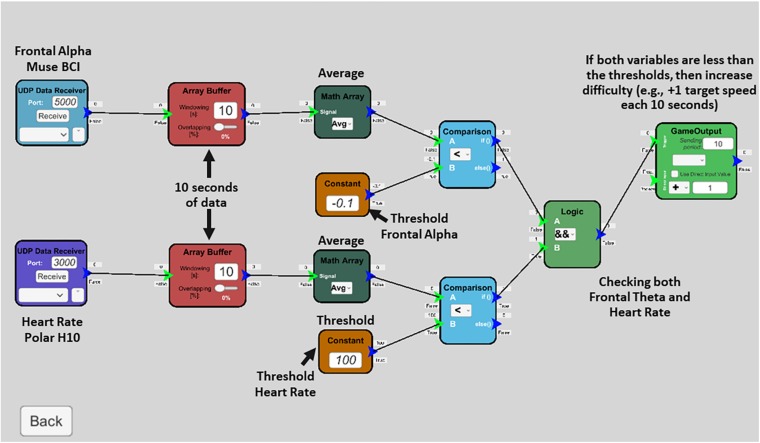
Adaptive rule created in the BL Engine based on the psychophysiological model.

The simulation difficulty in the BioPhyS covered easy, medium and hard scenarios. The design of those scenarios intended to evoke responses from police officers during specific human states such as minimal engagement (simple challenge considering their skills), engagement (balanced challenge/skill), and frustration (tough challenge considering their skills). The captured neurophysiological and cardiovascular responses from police officers across the simulation difficulty levels reflect specific physiological signatures that must be used to define the automatic adaption intended. Firstly, two specific EEG metrics showed statistical significance across simulation difficulties, frontal theta and theta/beta ratio. However, for real-time adaptation, frontal theta is preferred since it can be captured directly from the sensor without any further computation effort (e.g., dividing one bandpower by other). Secondly, cardiovascular responses also revealed significant differences in HR and RMMSD and VLW for HRV. HR is also preferred considering computational efficiency issues for real-time adaptation. Additionally, one of the firearms instructors of the research team pointed out literature suggesting specific sympathetic responses that are desired for trainees to elicit during the training. Literature research revealed the 100–115 BPMs range as the targeted HR zone defined by firearm trainers to stress the heart enough to facilitate timely cognitive responses without hampering fine motor skill performance (Mason, 1998). Therefore, we used frontal theta as concentration (mental focus) and HR as calmness (cardiovascular regulation) metrics to create an adaptive rule in the BL Engine. The rule is shown schematically in Figure 7. The implementation allows the rules to be easily modified by dragging-and-dropping specific blocks that allow connecting logical instructions.

**FIGURE 8:**
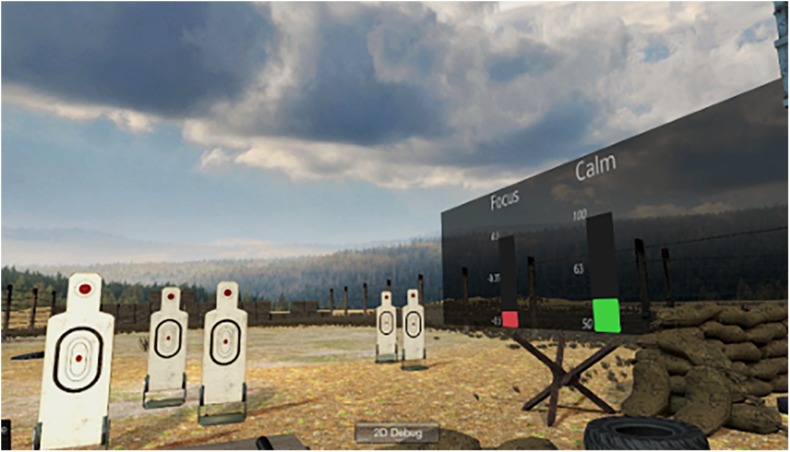
Integration of the BioPhyS and the BL Engine showing how it can be used to provide biofeedback based on the calmness and focus metrics.

The rule involves capturing physiological data from data receiver blocks and averaging it using the array buffer and math array blocks. Averaged physiological data is then compared against the threshold values that are inferred from the psychophysiological model to finally create the changes in the simulation based on the detected values. For instance, this rule creates modulations toward increasing simulation difficulty (e.g., increasing targets speed and reducing target size) if police officers are not reaching the intended targeted values for getting them engaged (e.g., −0.1 dB frontal alpha and 100 BPMs for HR). Additionally, data from the BL Engine can be sent to the BioPhyS to provide a biofeedback display (panel at the right side of Figure 8) for the police officers and investigate the effects of implicit and explicit feedback on training performance (Kuikkaniemi et al., 2010).

Although controlled experiments to validate the real-time model have not been carried out yet, the proposed model and computational solution serve as an initial asset for the future integration of biocybernetic adaptation.

## Discussion

We present a repeated-measures experiment of firearms training using an HMD-VR setup and neurophysiological and cardiovascular measurements to model the psychophysiological responses of 10 (*n* = 10) police officers to different simulation difficulty levels. To induce specific graded stressors in the simulation, the system was designed to manually modulate specific parameters in the VR simulator (e.g., target speed). Three different scenarios (*easy*, *medium*, and *hard*) were used to investigate the police officer’s physiological and subjective responses while actively shooting in the simulation and results were compared including measurements from resting states. Results show how police officers’ brainwaves and cardiovascular responses were significantly affected by modulations in the simulation difficulty. Particularly, frontal theta values were significantly different between *easy* and *hard* difficulty levels whereas HR and HRV data (RMSSD and VLF) were also significantly different during resting once compared with the *easy*, *medium*, and *hard* difficulties.

### Police Officers and Cognitive Readiness

Crucial elements for police firearms training include mastering very specific psychophysiological responses associated with controlled breathing while shooting. Novel immersive VR systems as the BioPhyS, allow the recreation of realistic scenarios where trainees can be exposed to different stressors while physiological responses are recorded. We showed how frontal theta values were significantly higher during the simulation difficulties that were not created to frustrate the police officers. Decrements in theta (θ) values have been commonly associated with non-attentional states such as drowsiness, severe sleepiness (Liu et al., 2009), or lower levels of arousal (Sanei and Chambers, 2013). Thus, controlled modulations toward increasing frontal theta (θ) levels can help police officers to train cognitive readiness during shooting scenarios. Moreover, values of the theta/beta ratio collected during the experiment showed that this metric could be used to differentiate between difficulty levels in simulations for firearm training.

On the other hand, police shooters are affected not only by responses of the central nervous system, but also the peripheral system as well. A consistent and mastered skill of controlling cardiovascular responses able to produce desired patterns of respiration has been identified as a major component of firearms training (Johnson, 2007). HR levels significantly increased from the resting or *baseline* recording where police officers were not engaged with shooting activities. A trend in Figure 4 shows how HR levels were incrementally impacted by the simulation difficulty, demonstrating how via systematic modulations in the simulation parameters, cardiovascular responses of shooters can be affected. Similar responses have been found in police officers after training practices under pressure (Oudejans, 2008). Although only few parameters were used to modulate the simulation difficulties, further research using more metaphorical simulation variables (e.g., daylight and rain intensity) could reveal the ideal configuration to elicit the desired cardiovascular responses (Marchiori et al., 2018). Finally, we believe the overall user experience was positively perceived by police officers due to (i) the wearability of the physiological sensors which avoided extra discomfort produced by more invasive setups (e.g., multi-channel ECG signals or EEG caps), (ii) the realism of the simulation provided by the VR equipment used as well as the accuracy of the motion tracking achieved through the room-scale play area using two lighthouses, and (iii) the usefulness of the system perceived by the instructors and other police officers who were enthusiastic about using this technology in real training.

### Characterization, Psychophysiological Model, and the Physiological Intelligence Layer

The characterization study with 10 police officers serves as a starting point for the design of physiological computing system that would be able to create the biocybernetic adaptation in the BioPhyS. From the model (see Figure 6) two trends can be identified, while HR levels increased proportionally with the simulation difficulty, the frontal theta values decreased. Theta waves have been found as a sensitive neurophysiological marker to describe participant’s discomfort (Heo and Yoon, 2020) and motion sickness (Park et al., 2008) in gaming/VR related studies. Moreover, cardiovascular responses such as HR levels have been also studied in firearms training showing heightened states of arousal after active shooting (Heim et al., 2006). Thus, differences between the active shooting and baseline revealed that just by engaging people in the virtual shooting activity without considering the difficulty level, specific psychophysiological patterns can be identified. This is important since it allows differentiating between the physiological signature of being wearing the headset and sensors as well as being standing holding the controllers and the cognitive and cardiovascular cost of shooting. In other words, this provides a certain level of context awareness, so the adaptive system would be able to create more personalize modulations (Novak, 2014). Finally, the adaptive rule created using the BL Engine tool (see Figure 7) shows how to move from the theoretical model to a real software implementation by: (i) combining both cardiovascular and neurophysiological features into a clearly defined and transparent adaptive rule, (ii) allowing the modifications of the rule and values in real-time, so speeding up threshold’s adjustment for real-time adaptation, and (iii) fully integrating the VR simulator with the BL Engine, so a bidirectional communication will take place enabling research on biofeedback visualization strategies (Kuikkaniemi et al., 2010). Relatedly, Kuikkaniemi et al. (2010) hypothesized that the increase in immersion due to adaptive biofeedback that they demonstrated in the context of first-shooter games could also be achieved in other contexts such as 3D virtual environments (Malińska et al., 2015). There is also evidence that neurofeedback, which BioPhyS employs, may boost the effects of cognitive training (Dessy et al., 2018). Biocybernetic adaptation integrated during exercise-based videogames called Exergames was used effectively to encourage older users to exert in targeted cardiac zones (Muñoz et al., 2018). A similar concept can be explored here, where the difference between the trainee’s HR response and the target or setpoint HR can be used to drive attributes of the simulation task, e.g., target speed or hindering rain intensity, which, in turn, would be expected to drive the trainee’s HR.

### Future Work With Virtual Reality and Biocybernetic Adaptation

In the BioPhyS system programmed for self-regulation training, attributes of the VR simulation task, e.g., target speed or rain intensity, are adjusted in ways designed to encourage certain psychophysiological response changes and to discourage others. Physiological self-regulation training with the BioPhyS leverages two memory process principles to promote the transfer of skill learning. Stimuli in the VR environment are designed to match stimuli in the real-world shooting situation, thereby promoting the transfer of firearm skill learning via a memory process known as encoding specificity (Pugnetti et al., 2001; Jaiswal et al., 2010). Physiological states that are determined to be effective in the real-world shooting situation are rewarded in the training setting by biocybernetic adaptation, thereby promoting transfer of learning via a memory process known as state-dependent learning (Pugnetti et al., 1996). In order to investigate how to integrate biocybernetic adaptation strategies into novel immersive systems for firearm training, the BioPhyS can leverage physiological computing technologies to be empowered with intelligent capabilities. Specifically, in this project, the BL Engine offers strategies suitable for enhancing shooting performance by controlling the elements in the virtual environment that affect the simulation difficulty and the officer’s concentration and relaxation levels (concentrated and so-called “calm, cool, and collected” state). To improve their scores in the shooting scenarios, police officers would have to use self-regulation strategies (e.g., respiration and attentional control) that will keep them calm and focused, hence reducing the probability of making mistakes in real-life situations (Blacker et al., 2018). Additionally, the physiological challenges can be modified (in real-time, if needed) by trainers allowing a very dynamic and personalized training.

Including physiological sensing technologies in novel immersive and interactive technologies such as augmented or VR has been posed as the new generation of gaming interfaces (Pope et al., 2014). This integration is far from being simple and straightforward since including the human in the loop carries several complexities widely discussed (Novak, 2014). Nevertheless, current advances in sensing technologies, game engine software, and hardware accessibility have allowed the development of very sophisticated *biocybernetically* adaptive systems empowered with the *immersivity* and realism of 52 (Kosunen, 2018; Muñoz et al., 2019). In order to get a more solid and integrated VR-biocybernetic confluence, advances in three aspects are required:

• Improvements in the hardware integration: to aid the design and development process of *biocybernetically* empowered VR applications, the integration of physiological sensing devices, and VR headsets should be minimally intrusive and less cumbersome. Although in our experience we used minimally intrusive sensors, the use of VR headsets that include sensors to record body signals (e.g., Neurable,^3^ Looxidlabs,^4^ Interaxon; Aimone et al., 2018) will aid the widespread of such technological symbiosis.
• Advances in the software intelligence: even though several good examples of using biocybernetic adaptation in games implemented simplistic adaptive techniques to maximize the desired target psychophysiological state (Pope et al., 2014; Ewing et al., 2016), more sophisticated adaptive algorithms can be used to provide a more robust and personalized experience. For instance, techniques based on control feedback principles such as PID controllers can be used to create more accurate modulations in the simulation variables, thus facilitating a faster and more sustained “desired” physiological response (Parnandi and Gutierrez-Osuna, 2014; Muñoz et al., 2019). Machine learning techniques can also be used to detect complex psychophysiological states such as stress or workload and adapt the content accordingly (Panicker and Gayathri, 2019).
• Spreading out successful use cases outside academia: progress in *biocybernetically* adaptive technologies has been mostly presented in academic environments with only few exceptions (Pope et al., 2014). Although this construct has shown clear evidence of how it can be used in scenarios like gaming, healthcare, and training (Fairclough, 2017), examples of successful and *commercializable* use cases of *biocybernetically* empowered products are still scarce.
• Considerations for simulator and VR sickness are vital for meaningful results to be gleaned from this and similar research embedding physiological computing into simulated (VR, AR, MR, etc.) experiences/environments. Metrics for the effects that simulated stimuli have on test subjects have been developed and examined extensively (Kennedy et al., 1993, 2001; Saredakis et al., 2020) and should be applied in future studies involving similar technologies.

## Study Limitations

Our study encloses numerous limitations that are listed as follows:

• Sample size: the size of the sample, *n* = 10, was small. Therefore, caution should be exercised in generalizing the results of this VR characterization study. The results should be confirmed in future studies with a larger sample size. Additionally, participants were diverse in terms of training skills and demographics.
• Validation of the physiologically adaptive model: although both the psychophysiological model (Figure 6) and the adaptive rule created in the BL Engine (Figure 7) serve as valuable inputs for the real-time biocybernetic loop implementation, a controlled experiment validating the proposed methods is needed to better understand the effects of physiological adaptation to train self-regulation skills in police officers. Data variability, for instance, is a major challenge for implementing the real-time model since differences in physiological responses between resting states and active shooting differ considerably. For instance, HR differences are largely the same in all simulation difficulties whereas frontal theta only showed differences between easy and hard difficulties.
• Limitations of the EEG sensor: the EEG wearable had few electrode sites, limiting EEG data density. Furthermore, motion artifact in the EEG and ECG signals can be an issue with an active VR task such as target shooting. Nevertheless, informative studies employing these three technologies have been successfully conducted (Marín-Morales et al., 2019).

• Exposure time for each training scenario: a short duration, 3 min, was the time used to record the physiological responses during the pre-established training scenarios (*easy*, *medium*, and *hard*), which should be analyzed carefully since firearms training sessions could last more time (Johnson, 2007). However, it is worth mentioning that critical incidents can last seconds and training in physiological regulation could play an important role in preparing police officers for such scenarios. Besides, it is also important to consider that extracting cardiovascular and neurophysiological features in short time intervals (e.g., 1–5 min) could bring more accurate information of the specific psychophysiological responses rather than averaging an entire, long session (e.g., more than 5 min).
• Limitations in the weapon used: due to technical issues in integrating more realistic air-pistols in our study, we used the standard controllers of the VR equipment which can be seen as less accurate in terms of creating a vivid simulation. Also, the use of the virtual revolver differs from the pistols used in real scenarios, thus can be seen as an extra limitation.

Further research is needed to provide a more conclusive understanding of how biocybernetic adaptation can impact police officers’ performance in firearms training using VR simulations.

## Conclusion

This study characterized the dynamical neural and cardiac responses of police officers in a VR based target shooting scenario and provides the foundation for a biocybernetic system with an intelligent adaptation layer. The simulation difficulty factor was shown to influence shooting performance, frontal theta brainwave activity and HR, providing a specification of values of the physiological variables to be targeted in a biocybernetic adaptation system as well as to serve as a control measure for assessing the effects of adding a biocybernetic adaptation layer to a VR training system. Assessment of the results from the Simulator Sickness Questionnaire showed that the experience was generally well tolerated. Further development and refinement of the VR simulation capability (including ergonomic improvements to the headset) are suggested to mitigate unintended effects of simulation stimuli on users of such systems. An explanation was given for employing the neural and cardiac findings to integrate physiological intelligence into the VR simulator using a specialized software tool, the BL Engine. Next steps on this research must employ the provided model to quantify the effects of using biocybernetic adaptation in VR-based firearms training for police officers.

## Data Availability Statement

The datasets generated for this study are not publicly available as participants did not provide their informed consent for the public sharing of their data. The datasets are available on request to john.munoz.hci@uwaterloo.ca.

## Ethics Statement

Ethical review and approval was completed for the study on human participants in accordance with the local legislation and institutional requirements. The patients/participants provided their written informed consent to participate in this study. Written informed consent was obtained from the individual(s) for the publication of any potentially identifiable images or data included in this manuscript.

## Author Contributions

JM and AP designed and defined the BioPhyS approach. JM conducted the experiment and collected the data. LQ developed the VR simulation and carried out the integration with the BL Engine. CS contributed and reviewed the manuscript. All authors revised and approved the current version of the manuscript.

## Conflict of Interest

AP was employed by the company Learning Engagement Technologies.

The remaining authors declare that the research was conducted in the absence of any commercial or financial relationships that could be construed as a potential conflict of interest.

## Footnotes

1 https://sites.google.com/a/interaxon.ca/muse-developer-site/muselab

2 https://support.polar.com/en/support/Selection_Info_Analysis_for_R_R_data

3 http://www.neurable.com/

4 https://looxidlabs.com/
